# Lifestyle factors and DNA methylation-based aging clocks: cross-sectional and longitudinal associations in the Singapore diet and healthy aging cohort

**DOI:** 10.1016/j.tjpad.2026.100522

**Published:** 2026-02-27

**Authors:** Jiatong Shan, Jian Hua Tay, Kaisy Xinhong Ye, Jiuyu Guo, Luwen Cao, Yan Zeng, Tih-Shih Lee, Kua Ee Heok, Brian K. Kennedy, Andrea B. Maier, Lei Feng

**Affiliations:** a Healthy Longevity Translational Research Programme, Yong Loo Lin School of Medicine, National University of Singapore, Singapore 117456, Singapore; b Centre for Healthy Longevity, National University Health System, Singapore 117456, Singapore; c Department of Psychological Medicine, Yong Loo Lin School of Medicine, National University of Singapore, Singapore 119007, Singapore; d NUS Academy for Healthy Longevity, Yong Loo Lin School of Medicine National University of Singapore, Singapore, Singapore; e School of Medicine, Wuhan University of Science and Technology, China; f Duke-NUS Signature Research Programme in Neuroscience & Behavioural Disorders, Singapore; g Department of Human Movement Sciences, @AgeAmsterdam, Faculty of Behavioral and Movement Sciences, Vrije Universiteit Amsterdam, Amsterdam Movement Sciences, Amsterdam, the Netherlands; h Department of Physiology, Yong Loo Lin School of Medicine, National University of Singapore, Singapore 117593, Singapore; i Department of Biochemistry, Yong Loo Lin School of Medicine, National University of Singapore, Singapore 117596, Singapore

**Keywords:** Lifestyles, Epigenetic age, Cross-sectional, Longitudinal, Older adults

## Abstract

**Background:**

Lifestyle factors play a critical role in healthy aging, yet their relationships with aging biomarkers remain insufficiently characterized, particularly in Asian populations. This study aimed to examine the cross-sectional and longitudinal associations between 15 modifiable lifestyle factors and two DNA methylation (DNAm) clocks (GrimAge acceleration [AgeDev] and DunedinPACE) in a cohort of older Asian adults.

**Methods:**

We conducted a cross-sectional analysis of 631 participants (median age 70.0 years; 72.6% female) and a longitudinal analysis of 114 participants (mean follow-up 3.96 years) from the Singapore Diet and Healthy Aging (DaHA) cohort. Lifestyle exposures were assessed using validated self-administered questionnaires. Peripheral blood DNAm profiles were generated using the Illumina MethylationEPIC array. Multivariable linear regression models were applied to evaluate associations between lifestyle factors and DNAm clocks, adjusting for sociodemographic covariates, health status, and immune cell-type proportions.

**Results:**

In cross-sectional analyses, smoking history showed robust positive associations with accelerated epigenetic aging (GrimAge AgeDev: β = 1.45, 95% CI 1.13–1.77, *p* < 0.0001; DunedinPACE: β = 0.63, 95% CI 0.22–1.05, *p* = 0.003). Conversely, weekly physical activity was associated with slower aging (GrimAge AgeDev: β = –0.22, 95% CI –0.40 to –0.04, *p* = 0.02), as was daily engagement in cognitively stimulating activities (GrimAge AgeDev: β = –0.16, 95% CI –0.31 to –0.01, *p* = 0.04). Weekly feelings of stress were initially associated with greater GrimAge AgeDev, but this relationship was attenuated after full adjustment. No significant longitudinal associations were detected, which may reflect limited statistical power and the stability of long-standing lifestyle behaviors over the follow-up period.

**Conclusions:**

These findings highlight significant cross-sectional associations between key modifiable lifestyle factors, particularly smoking, physical activity, and cognitive engagement, and epigenetic aging in an older Asian cohort. The results suggest that interventions targeting these behaviors may modulate the pace of biological aging. The absence of significant longitudinal associations underscores the need for larger prospective studies with longer follow-up and continued validation of epigenetic clocks in diverse populations to confirm these relationships over time.

## 1 Introduction

Lifestyle factors profoundly influence long-term health and longevity. Epidemiological studies have linked specific behaviors to a wide array of age-related outcomes, including physical functioning, cognitive decline, chronic diseases, and mortality [[1], [2], [3]]. Physical activity demonstrates robust inverse associations with risks of type 2 diabetes and cardiovascular events, and a modest increase in physical activity replacing sedentary time can yield a significant health benefit [4]. Tobacco smoking shows strong association with reductions in both total and disability‐free life expectancy [5]. Regular engagement in cognitive activities slows cognitive decline and lowers dementia risk [6]. The 2024 Lancet Commission on dementia prevention identified 14 potentially modifiable risk factors, such as physical inactivity, smoking, harmful alcohol use, obesity, diabetes, depression, and social isolation [7], and estimated that addressing these factors could prevent or delay approximately 45 % of dementia cases globally. Building on this life-course framework, the present study examines 15 specific, day-to-day lifestyle behaviors that are directly modifiable at the individual level and theoretically aligned with these risk domains. These behaviors span physical, social, cognitive, and religious activities; light exposure; sleep–wake patterns; work hours; oral and bowel habits; tobacco use; alcohol intake; and perceived stress. Collectively, adherence to healthy lifestyle behaviors represents a promising approach to promote healthy longevity.

Deoxyribonucleic acid (DNA) methylation (DNAm)-based clocks, referred to as epigenetic clocks, are biomarkers of ageing based on methylation at cytosine-phosphate-guanine (CpG) sites within the genome. First-generation clocks, including Horvath [8] and Hannum [9], were trained to predict chronological age [10]. Second-generation clocks (PhenoAge [11], GrimAge [12], GrimAge2 [13]) were trained against clinical phenotypes and mortality risk [4,11,12]. Third-generation clocks, including DunedinPoAm [14] and DunedinPACE [15], were calibrated against longitudinal multi-system physiological changes. Given the development of these sophisticated ageing biomarkers, a growing body of research has demonstrated associations between lifestyle-related factors and accelerated or delayed DNAm age deviation (AgeDev), which is the difference between biomarker-predicted biological age and chronological age [16]. A review [17] showed that regular exercise and physical activity are consistently associated with lower GrimAge AgeDev in 34,710 participants of European ancestry [18], 50,884 women recruited from the United States and Puerto Rico [19], and 5209 participants from the Framingham Heart Study (FHS) [20]. Smoking is associated with higher GrimAge AgeDev as well as the pace of aging in DunedinPACE in Health and Retirement Survey (HRS) [21] and Add Health cohort [22]. In general, GrimAge was shown to outperform the first-generation clocks in predicting both morbidity and mortality [23], and identifying age-related decline in the clinical phenotypes [24]. DunedinPACE showed high test-retest reliability and in analysis of morbidity, disability, and mortality, it even added incremental prediction beyond GrimAge [15], which is promising to advance precision medicine [15].

Despite these findings, most studies only focused on a single lifestyle, and predominantly in cohorts of non-Asian ancestry. The associations between lifestyle factors and DNAm clocks among Asian older adults remain largely unexplored. The present study addresses these gaps by investigating the associations between 15 modifiable lifestyle factors and DNAm clocks in the Diet and Healthy Ageing (DaHA) cohort of older Asian adults.

## 2 Method

### 2.1 Study participants

Participants were recruited from the DaHA cohort study in Singapore through door-to-door invitations. Between 2011 and 2017, DaHA recruited 1060 individuals aged 60 years or older. During the follow up period from 2017 to 2020 (approximately five years after baseline), 620 participants underwent reassessment. The NUS Institutional Review Board approved the study [25] and all participants provided written informed consent.

### 2.2 Lifestyle factors assessment

To comprehensively evaluate lifestyle behaviors, fifteen lifestyle factors were ascertained through a self-reported survey: participating in (1) physical activities, (2) social activities, (3) cognitively demanding activities, (4) religious/spiritual activities, as well as (5) sunlight exposure, (6) brushing teeth, (7) stopping eating before feeling full, (8) sleeping less than 6 h/day or (9) more than 9 h/day, (10) feeling sleepy during the day, (11) having a bowel movement within 10 min, (12) working more than 9 h/day, (13) cigarette smoking, (14) alcohol consumption, and (15) feeling stressed. These 15 items were selected to capture modifiable lifestyle behaviors across multiple domains: activity engagement (physical, social, cognitive, religious), light and sleep-wake habits, self-care routines (oral hygiene and bowel regularity), work patterns, substance use, and perceived stress. Each item corresponds to behaviors previously treated as lifestyle or health-related habits in epidemiologic or psychological research, even though no prior study has combined exactly this set of 15 behaviors into a single composite index [[26], [27], [28]]. Cigarette smoking history was classified participants as never, and ever smokers (≥100 lifetime cigarettes as threshold) [29]. Alcohol consumption was categorized as never and ever current drinkers (≥12 drinks in lifetime as threshold) [30], given the majority of participants were females. Participating in cognitively demanding activities was defined as any participation in tasks that require a high level of mental effort and complexity, challenging individuals to use higher-order thinking skills like analysis, evaluation, and synthesis to understand and solve problems [31] such as doing puzzles, Chinese calligraphy, reading, playing chess. Participants reported the frequency of each behavior using a five-point ordinal scale: “never or rarely”, “more than once a month but less than once a week”, “1–3 times a week”, “4–6 times a week”, and “daily”. Frequencies were transformed into three dummy categorical variables: less than weekly (reference category), weekly, and daily [32].

### 2.3 DNAm clock analysis

Fasting venous blood was drawn from each participant, and genomic DNA was isolated from buffy coat. DNA methylation was profiled on the Infinium MethylationEPIC v1.0 Beadchip (Illumina Inc., San Diego, CA). GrimAge and DunedinPACE were calculated. Raw β values were pre‐processed and normalized using SeSAMe v1.24.0′s “openSesame” pipeline, applying Noob background correction, dye‐bias normalization (DyeBiasNL), and pOOBAH probe filtering. Missing values were then imputed with imputeBetas() [33]. Age estimates were generated with the pyageing v0.1.11 package under default settings [34].

### 2.4 DNAm-based cell composition analysis

To infer immune‐cell composition, β values were analyzed by Robust Partial Correlations in EpiDISH v2.22.0 using the cent7CT.m reference [[35], [36], [37]], yielding seven epi7 variables corresponding to the following cell-type signatures: B cells, natural killer cells, CD4⁺ T cells, CD8⁺ T cells, monocytes, neutrophils, and eosinophils [35]. Principal component analysis (PCA) of the 7-cell composition was conducted, and principal components (PCs) capturing 90 % of the variance were used in statistical analysis to avoid co-linearity [38].

### 2.5 Statistical analysis

All statistical analyses were carried out in R 4.4.2. Descriptive data are reported as mean ± standard deviation (SD), median and interquartile range (IQR), or number and percentage ( %). Missing data were omitted without imputation. For cross‐sectional associations, linear regression models were fitted with GrimAge AgeDev or DunedinPACE as outcomes and each of the 15 lifestyles as predictors.

Model 1 was for sex, while model 2 further fully adjusted for cigarette smoking, alcohol consumption, body mass index (BMI, weight/height^2^), education status and the PCs of 7 cell compositions. When the count for any category was below 10, the category with largest count was set as the reference group and the remaining categories were combined [39].

Only individuals who had both timepoints were included to adjust for intra-person effects, reducing the total number to 114 participants. Longitudinal Δ-AgeDev was defined as the 4-year change in AgeDev between visits, standardized by baseline SD: Δ-AgeDev = (AgeDev t2 – AgeDev t1)/ σ_AgeDev t1_, where t2 represents follow-up and t1represents baseline [25]. After standardisation (z‑scoring), each Δ‑clock had a mean of 0 and SD of 1 by design. We performed a linear regression with Δ‐AgeDev as the dependent variable and baseline lifestyle factors, including baseline AgeDev, chronological age, and the same covariates described above as independent variables. Results are reported with 95 % confidence intervals (CI) and nominal P-values.

## 3 Results

**Fig. 1:**
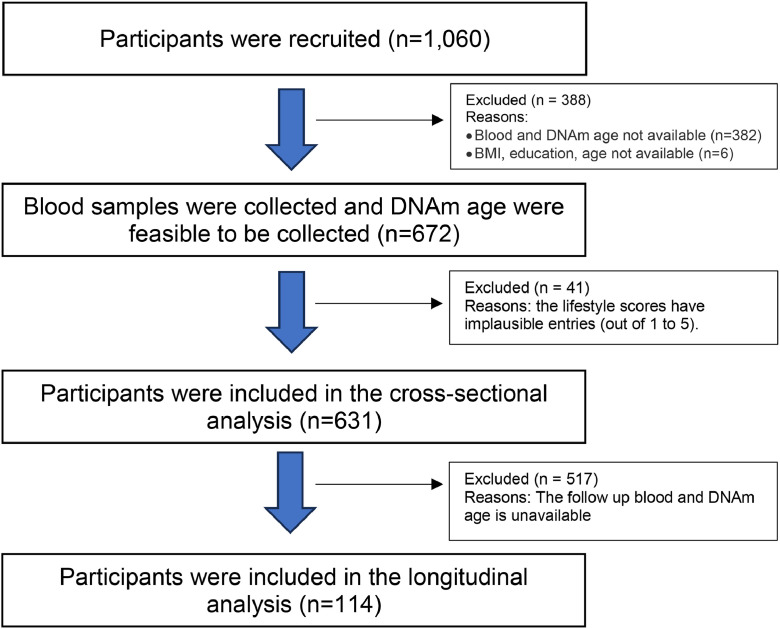
The flowchart of participants included in this study.

Table 1 shows the characteristics of participants in the DaHA cohort. Of 631 participants (median age 70.0 years, IQR [66.0, 74.0]), 458 (72.6 %) were female and 608 (96.4 %) were of Chinese ethnicity. The median body mass index (BMI) was 24.0 kg/m² (IQR [21.8 26.4]). Mean PCGrimAge was 77.6 years (SD 5.4) and mean DunedinPACE score was 1.11 (SD 0.11). Fig. 1 is the flowchart of participants included in this study.

**Table 1:** Characteristics of baseline of DaHa cohort cross-sectionally (*n* = 631).

Characteristic	Value
**Demographics and health status**	
Age, year	70.0 [66.0, 74.0]
Female, *n* ( %)	458 (72.6)
Race, *n* ( %)	
Chinese	608 (96.35)
Others including Indian and Malays	21 (3.32)
At least primary school education, *n* ( %)	408 (64.66)
Body Mass Index, kg/m^2^	23.98 [21.82, 26.41]
**DNAm clocks**	
PCGrimAge, years, mean (SD)	77.63 (5.40)
DunedinPACE, score, mean (SD)	1.11 (0.11)
**Lifestyle factors**	
Participate in physical activities, *n* ( %)	
less than weekly	88 (13.95)
weekly	304 (48.18)
daily	239 (37.88)
Participate in social activities, *n* ( %)	
less than weekly	172 (27.26)
weekly	376 (59.59)
daily	83 (13.15)
Participate in cognitively demanding activities, *n* ( %)	
less than weekly	183 (29.00)
weekly	200 (31.70)
daily	248 (39.30)
Participate in religious / spiritual activities, *n* ( %)	
less than weekly	427 (67.67)
weekly	169 (26.78)
daily	35 (5.55)
Expose yourself to sunlight, *n* ( %)	
less than weekly	38 (6.02)
weekly	190 (30.11)
daily	403 (63.87)
Brush your teeth, *n* ( %)	
not daily	5 (0.79)
daily	626 (99.21)
Stop eating before feeling full, *n* ( %)	
less than weekly	142 (22.50)
weekly	135 (21.39)
daily	354 (56.10)
Sleep less than 6 h / day, *n* ( %)	
less than weekly	442 (70.05)
weekly	119 (18.86)
daily	70 (11.09)
Sleep more than 9 h / day, *n* ( %)	
less than weekly	581 (92.08)
weekly	24 (3.80)
daily	26 (4.12)
Feel sleepy during the day, *n* ( %)	
less than weekly	299 (47.39)
weekly	241 (38.19)
daily	91 (14.42)
Have a bowel movement within 10 min, *n* ( %)	
less than weekly	64 (10.14)
weekly	107 (16.96)
daily	460 (72.90)
Work more than 9 h / day, *n* ( %)	
never or rarely	600 (95.09)
ever	31 (4.91)
Smoke cigarette, *n* ( %)	
never or rarely	607 (96.20)
ever	24 (3.80)
Drink alcohol, *n* ( %)	
never or rarely	569 (90.17)
ever	62 (9.83)
Feel stressed, *n* ( %)	
less than weekly	546 (86.53)
weekly	71 (11.25)
daily	14 (2.22)

Data indicate as median [IQR] unless indicated otherwise. Abbreviations: IQR = interquartile range; SD = standard deviation.

### 3.1 Cross-sectional analysis

**Fig. 2:**
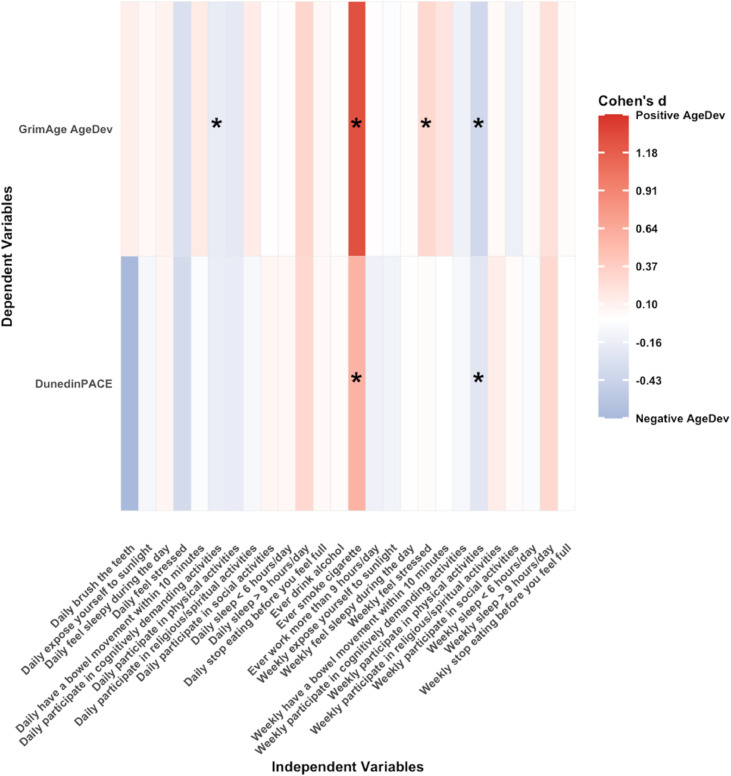
Cross-sectional associations of 15 lifestyles and GrimAge AgeDev and pace of ageing adjusted for age and sex. Each cell coloured in blue (negative association) or red (positive association) respectively. Statistically significant associations (*p* < 0.05) are indicated by asterisks within each cell. Abbreviations: AgeDev: Age devtion. Note: Lifestyles are categorical variables, and its effect size is β [95 % CI] (Δ‐clock SD compared to the reference group).

**Fig. 3:**
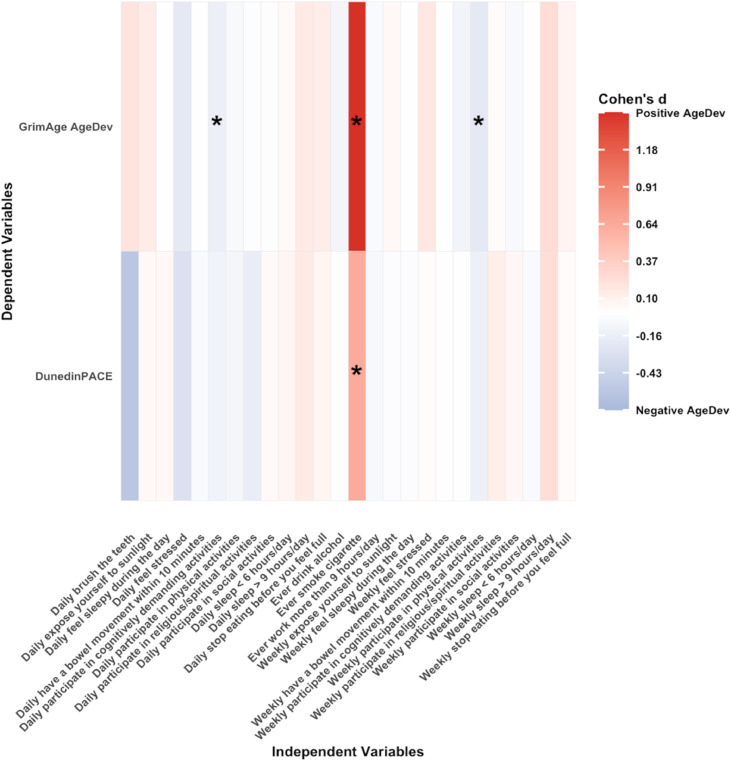
Cross-sectional associations of 15 lifestyles and GrimAge AgeDev and pace of ageing adjusted for age, sex, smoking status, alcohol status, BMI, education, and PCA of 7 cell compositions. Each cell coloured in blue (negative association) or red (positive association) respectively. Statistically significant associations (*p* < 0.05) are indicated by asterisks within each cell. Abbreviations: AgeDev: Age deviation. Note: Lifestyles are categorical variables, and its effect size is β [95 % CI] (Δ‐clock SD compared to the reference group).

Fig. 2, Fig. 3 show heatmaps of associations between lifestyle factors and DNAm age in cross-sectional analyses for model 1 and model 2, respectively. Smoking history was consistently associated with accelerated ageing. The regression coefficient β between smoking and GrimAge AgeDev in model 1 was 1.27 (95 % CI 0.91–1.62, *p* < 0.0001) and was higher in model 2 (β 1.45, 95 % CI 1.13–1.77; *p* < 0.0001) after adjusting for multiple potential covariates. The association between smoking and DunedinPACE was also larger in fully-adjusted model 2 (β 0.63, 95 % CI 0.22–1.05; *p* = 0.003) compared with model 1 (β 0.58, 95 % CI 0.16–0.99; *p* = 0.01). Weekly participation in physical activity was inversely associated with GrimAge AgeDev in model 1 (β –0.39, 95 % CI –0.60 to –0.18; *p* < 0.001) and model 2 (β –0.22, 95 % CI –0.40 to –0.04; *p* = 0.02), and with DunedinPACE in model 1 (β –0.24, 95 % CI –0.48 to –0.01; *p* = 0.04), although the latter association did not remain significant after adjustment. Daily engagement in cognitively demanding activities was associated with lower GrimAge AgeDev in both model 1 (β –0.19, 95 % CI –0.36 to –0.02; *p* = 0.03) and model 2 (β –0.16, 95 % CI –0.31 to –0.01; *p* = 0.04). Weekly feelings of stress were associated with a higher GrimAge AgeDev (β 0.29, 95 % CI 0.07 to 0.51; *p* = 0.01), but this association was not significant in the model 2 (see Supplementary Tables 1 and 2).

### 3.2 Longitudinal analysis

**Fig. 4:**
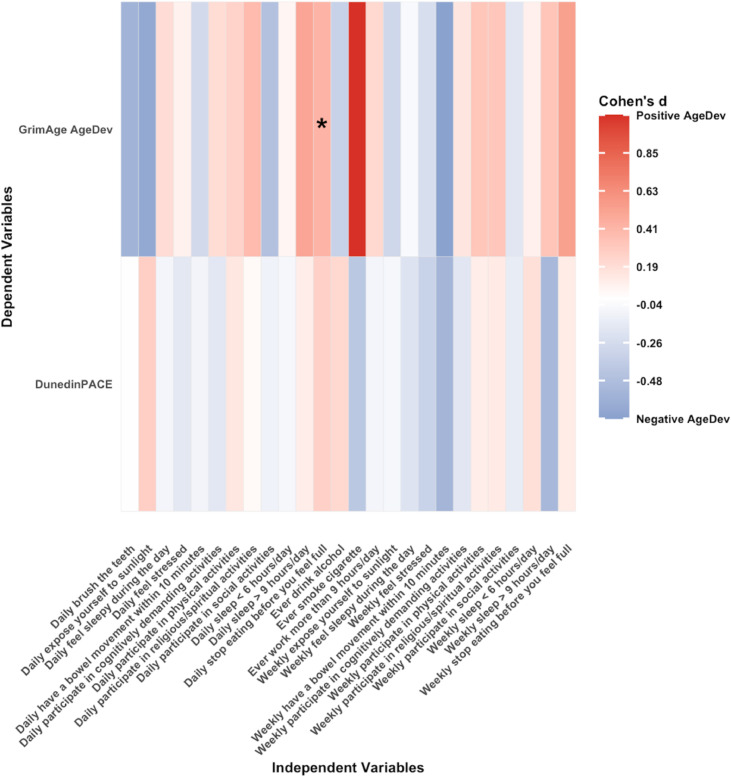
Standardised longitudinal associations between baseline lifestyle behaviors and change in GrimAge AgeDev and pace of ageing (Δ per SD), adjusting for baseline AgeDev and sex. Each cell displays the regression coefficient, coloured in blue (negative) or red (positive) respectively. Statistically significant associations (*p* < 0.05) are indicated by asterisks within each cell. Abbreviations: AgeDev: Age deviation. Note: Lifestyles are categorical variables, and its effect size is β [95 % CI] (Δ‐clock SD compared to the reference group).

**Fig. 5:**
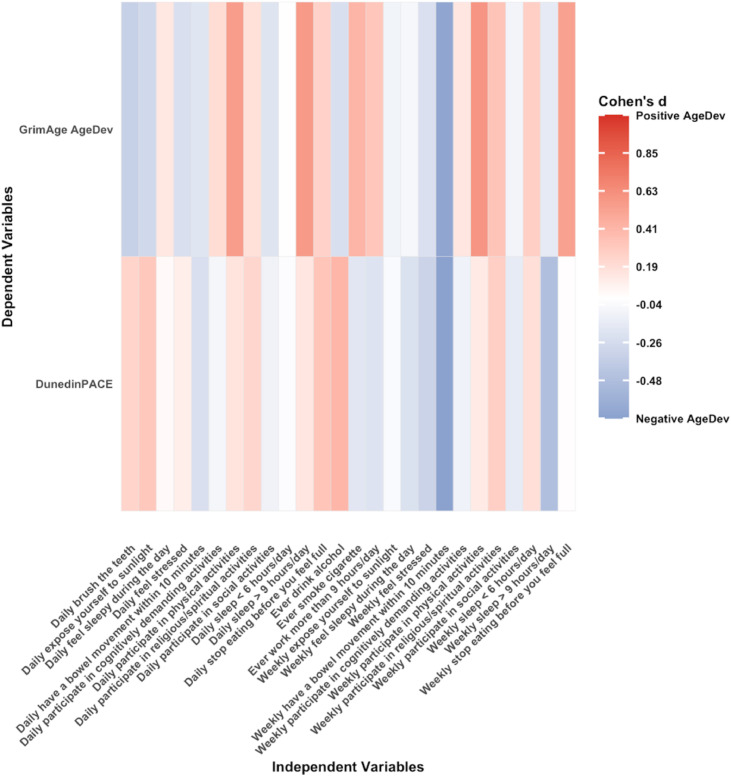
Standardised longitudinal associations between baseline lifestyle behaviors and change in GrimAge AgeDev and pace of ageing (Δ per SD), adjusting for baseline AgeDev, sex, smoking status, alcohol status, BMI, education and PCA of 7 cell compositions. Each cell displays the regression coefficient, coloured in blue (negative) or red (positive) respectively. Statistically significant associations (*p* < 0.05) are indicated by asterisks within each cell. Abbreviations: AgeDev: Age deviation. Note: Lifestyles are categorical variables, and its effect size is β [95 % CI] (Δ‐clock SD compared to the reference group).

The mean follow-up duration for longitudinal analysis was 3.96 (1.44) years. For longitudinal analysis (*n* = 114), the median (IQR) age was 66.0 [63.0, 72.0], 71.1 % female, 71.3 % were Chinese and 65.2 % having at least primary school education. The median (IQR) BMI is 23.2 kg/m² [21.5, 26.0]. Fig. 4, Fig. 5 show heatmaps of associations between lifestyle factors and DNAm age in longitudinal analyses for model 1 and full models, respectively. Stopping eating before feeling full on a daily basis was associated with accelerated GrimAge AgeDev in model 1 (β 0.42, 95 % CI 0.01–0.83; *p* = 0.049); however, this association was attenuated and lost significance after adjustment for BMI, education, cell composition, smoking, and alcohol use. As a sensitivity analysis, we additionally modeled stopping eating before feeling full as an ordinal trend term (0/1/2). The *P* for trend was non-significant across all specifications, suggesting that the observed associations are not well explained by a linear increase across frequency categories. No other lifestyle factors were significantly associated with GrimAge AgeDev, and no associations were observed with DunedinPACE (see supplementary Tables 3 and 4).

## 4 Discussion

### 4.1 Summary

In the DaHA cohort of older Asian adults, multiple lifestyle factors were observed to be correctionally associated with epigenetic measures of biological aging. Smoking history demonstrated the most robust and consistent association, being significantly linked with accelerated aging as measured by both GrimAge AgeDev and DunedinPACE in crude and fully adjusted models. Higher levels of physical activity and engagement in cognitively demanding activities were associated with decelerated aging, whereas weekly stress was positively associated with GrimAge AgeDev, although this association was attenuated after adjustment. Collectively, these findings underscore the relevance of modifiable lifestyle factors in influencing inter-individual variation in epigenetic aging within this cohort.

### 4.2 Biological underpinnings of GrimAge’s and DunedinPACE’s smoking associations

These findings align with evidence from other countries and age groups [40]. Cigarette smoke induces chronic inflammation and oxidative stress, which can accelerate breakdown of cellular homeostasis and lead to molecular ageing captured by certain DNAm clocks [21]. Smoking triggers persistent low-grade inflammation, characterized by elevated pro-inflammatory cytokines (e.g. interleukin-6 and TNF-α) and acute-phase reactants such as C-reactive protein (CRP) [41]. This inflammatory burden contributes to age-related tissue damage and functional decline [42]. Tobacco smoke also introduces reactive oxygen species that damage DNA and other macromolecules, potentially accelerating telomere shortening and prompting compensatory shifts in DNA methylation [43]. These biological pathways are directly reflected in clocks such as DunedinPACE and GrimAge, which were trained on markers of systemic inflammation [15], smoking exposure [13,14], and mortality risk [14], thereby embedding the molecular consequences of tobacco smoke in their predictions of biological ageing. Several studies have reported that cells exposed to tobacco smoke exhibit globally reduced 5-methylcytosine (5-mC) levels accompanied by increased 5-hydroxymethylcytosine (5-hmC) levels [[44], [45], [46]]. Heavy metals such as nickel and cadmium present in smoke impair the binding of DNA methyltransferases (DNMTs) to DNA, resulting in genomic DNA hypomethylation [46,47]. Additionally, ROS generated by cigarette smoke induce oxidative DNA lesions that interfere with normal methylation processes. For example, 8-oxo-2′-deoxyguanosine (8-oxodG) lesions at guanine within CpG dinucleotides significantly prevent methylation of the preceding cytosine [48]. Furthermore, tobacco smoke exposure can compromise DNA repair mechanisms, potentially leading to active loss of DNA methylation marks [49].

### 4.3 Biological underpinnings of GrimAge’s and DunedinPACE’s physical activity associations

Regarding physical activity, our results showed that weekly physical activity was significantly associated with lower GrimAge AgeDev, whereas daily physical activity did not reach statistical significance. Exercise-induced stress needs recovery, and this process may be blunted with excessive frequency, such as daily intense exercise, especially for older adults [50]. Accordingly, the WHO recommendation for older adults is much less than those for young and middle-aged adults [51]. However, because physical activity was captured using only three categorical response levels via face-to-face interview in our study, the findings also should be interpreted cautiously due to inadequate quantification of physical activities and potential information bias [52]. Objective measurements, such as accelerometers, should be used to gather more accurate data to confirm or repute the potential U shape association [53].

Our findings align with prior evidence suggesting that regular physical activity contributes to healthier biological aging. This effect may be mediated through beneficial alterations in DNA methylation across the genome, particularly at loci involved in metabolic regulation, oxidative stress response, tissue repair, and inflammation [54]. Additionally, regular activity counteracts immunosenescence, reduces chronic inflammation, and improves cardiovascular risk profiles, all of which mediate slower biological ageing [55]. The absence of association with DunedinPACE may be attributable to its validation framework, which emphasizes within-individual longitudinal change and midlife functional decline rather than prediction of mortality or lifestyle-related risk exposures [15]. By contrast, GrimAge was developed and validated in large, diverse epidemiological cohorts specifically to predict morbidity and mortality, and incorporates methylation surrogates for key risk factors (e.g., smoking, inflammatory proteins), rendering it more sensitive to cross-sectional associations such as those with physical activity [12].

### 4.4 GrimAge’s cognitive activity associations

Beyond physical activity, cognitive engagement also demonstrated protective associations with epigenetic ageing. Our findings that engagement in cognitively demanding activities is associated with a deceleration in GrimAge AgeDev are consistent with several studies [56,57]. Research has shown that individuals with poorer processing speed and working memory have higher GrimAge AgeDev, whereas those maintaining higher levels of cognitive activity exhibited slower ageing trajectories [56,57].

### 4.5 GrimAge’s stress associations

High perceived stress and stress-related disorders were shown to be associated with higher GrimAge AgeDev after adjusting for sex, consistent with prior reports. Greater cumulative lifetime stress exposure predicted significantly accelerated Horvath AgeDev after adjusting for health and lifestyle factors [58]. Additionally, women with chronic post-traumatic stress disorder (PTSD) have higher DunedinPACE compared to those without PTSD [59]. Individuals reporting high perceived stress levels tend to have elevated GrimAge age relative to chronological age, though results can vary with covariate adjustments [60]. More research on this psychological aspect is warranted given the lack of significance in our fully adjusted models.

### 4.6 Longitudinal findings

Our longitudinal analysis identified an unexpected association between eating behaviors and epigenetic aging. In this older cohort, daily practice of stopping eating before satiety was associated with accelerated epigenetic aging. This observation may not necessarily reflect deliberate caloric restriction but rather age-related physiological changes, such as diminished appetite, impaired gastrointestinal function, or underlying health conditions, which could increase the risk of undernutrition and contribute to accelerated physiological decline [61,62]. Undernutrition in late life has emerged as both a marker and potential driver of accelerated biological ageing [63]. A cross-sectional study of Americans aged over 50 found that moderate-to-severe malnutrition (assessed by nutritional risk indices) was linked to significantly higher GrimAge (approximately 4 years older than peers, *p* < 0.01) and elevated DunedinPoAm/PACE score, indicating faster ageing pace [64].

Notably, frailty assessments derived from objective physical performance measures indicated that 58 of 74 participants (78 %) who reported daily eating restraint met at least one criterion for physical frailty, defined as slow gait speed (>5 s over the test distance) or low mean hand grip strength (<18 kg). The high prevalence of frailty in this subgroup suggests that reduced food intake may reflect underlying health vulnerabilities, such as functional decline or age-related anorexia, rather than intentional caloric restriction, consistent with reports linking anorexia of aging with frailty and adverse functional outcomes [65]. Furthermore, frailty itself has been associated with epigenetic age acceleration in previous studies [66], raising the possibility that the observed association between daily eating restraint and accelerated epigenetic aging may be attributable to reverse causation or residual confounding. These findings underscore the need for caution in causal interpretation and highlight the importance of considering underlying health status when evaluating dietary behaviors in older populations. Although daily basis showed a significant DNAm age acceleration relative to less than weekly basis, the ordinal trend tests were not significant. This discrepancy is consistent with a potential threshold effect rather than a graded dose–response [67]. Given the relatively small sample size of the longitudinal subset, it is plausible that limited statistical power may have precluded the detection of significant associations across a broader range of measures. This consideration should be taken into account when interpreting these findings.

### 4.7 Different results in two biological clocks

In this study, we found that GrimAge shows greater sensitivity to lifestyle factors like cognitive activities and stress compared to DunedinPACE due to differences in the construction and training targets of these two clocks. GrimAge, a second-generation clock, predicts mortality risk using DNA methylation surrogates for plasma proteins (e.g., PAI-1, TIMP-1) and smoking pack-years, which reflect physiological impacts of lifestyle behaviors such as stress-induced inflammation or cognitive decline markers [68]. DunedinPACE, a third-generation pace-of-aging clock, tracks longitudinal changes in 20 biomarkers of organ function decline from the Dunedin Study, emphasizing genetic and developmental influences over modifiable lifestyle effects [15]. Studies indicate that GrimAge acceleration is associated with low education (proxy for cognitive activity), and pain (stress-related), and these associations are often less attenuated after lifestyle adjustment than those observed for other clocks [69,70]. In contrast, DunedinPACE shows weaker or more lifestyle-confounded associations with these factors, as BMI and smoking account for much of the observed links (e.g., effect sizes reduced by ∼50 % post-adjustment), consistent with its emphasis on pace rather than cumulative damage [71].

### 4.8 Different results in cross-sectional and longitudinal analyses

Several factors may explain why most associations observed cross-sectionally were not replicated longitudinally. The absence of statistically significant associations in the longitudinal analysis may be attributable to the relatively small number of participants with data available at both time points and the short duration of follow-up, which together limit the statistical power to detect changes over time. Furthermore, the lifestyle behaviors evaluated in this study largely represent long-standing habits rather than exposures newly adopted after baseline, indicating that participants may have already accrued most of the biological effects of these behaviors prior to the initial measurement. As a result, the between-wave differences in epigenetic aging are expected to be minimal, further reducing the likelihood of observing statistically significant longitudinal associations.

### 4.9 Strengths and limitations

The study has several key strengths. It includes a variety of lifestyles including activity, oral hygiene, sleep quality etc. in an older non-Western population. Furthermore, the integration of cross-sectional and longitudinal analyses provides a more comprehensive understanding of the associations (predictivity) of lifestyles and (in) DNAm age, by combining between-person differences with within-person change over time. Nevertheless, some limitations must be acknowledged. First, epigenetic clocks such as GrimAge and DunedinPACE may have limited generalizability to Asian populations, as both were trained primarily in European-ancestry cohorts [72]. This population shift, including differences in methylation quantitative trait loci (mQTLs), distinct lifestyle factors, batch effects, and leukocyte composition [73], can impair calibration and sensitivity when applied across populations. Enhanced validation among Asian cohorts is needed to improve fairness, robustness, and interpretability. Second, the ascertainment of lifestyles relied on participant self-report which may cause recall bias. Third, missing data were omitted from analyses without imputation as the data were missing completely at random.

### 4.10 Future direction

Future research should build upon the findings from this study. To ascertain the predictive ability of lifestyles for long-term DNAm age, a longer longitudinal follow-up of the DaHA group is necessary. In order to evaluate the wider transferability of physical activity and smoking, comparable validation studies in other significant populations, such as Malay and South Asian cohorts, are desperately needed given the ethnic variety of Asia. Lastly, lifestyle interventions should be tested in randomized controlled trials to establish their efficacy and confirm that they are clinically modifiable targets.

## 5 Conclusion

In this cohort of older Asian adults, smoking was robustly associated with accelerated biological aging as quantified by DNAm clocks, whereas engagement in physical and cognitively stimulating activities was associated with decelerated aging in cross-sectional analyses. These associations were not observed longitudinally, likely reflecting limited statistical power and the relative stability of lifestyle behaviors in late life. Collectively, these findings reinforce the importance of modifiable behavioral factors in shaping epigenetic aging trajectories within Asian populations and highlight the need for larger, long-term cohort studies and the development of ethnically calibrated biomarkers to elucidate causal relationships and inform targeted intervention strategies.

## Declaration of generative AI and AI-Assisted technologies

The authors declare that no generative AI or AI-assisted technologies were used in the preparation of this manuscript, including scientific writing, figures, images, or artwork.

## Data sharing

The data analysed are available from the corresponding author upon reasonable request by email.

## CRediT authorship contribution statement

**Jiatong Shan:** Writing – review & editing, Writing – original draft, Software, Methodology, Formal analysis, Data curation, Conceptualization. **Jian Hua Tay:** Software, Methodology, Formal analysis. **Kaisy Xinhong Ye:** Writing – review & editing, Supervision, Resources. **Jiuyu Guo:** Writing – review & editing, Data curation. **Luwen Cao:** Writing – review & editing. **Yan Zeng:** Writing – review & editing. **Tih-Shih Lee:** Writing – review & editing. **Kua Ee Heok:** Writing – review & editing. **Brian K. Kennedy:** Writing – review & editing. **Andrea B. Maier:** Writing – review & editing. **Lei Feng:** Writing – review & editing, Supervision, Resources, Investigation, Funding acquisition, Conceptualization.

## Declaration of competing interest

The authors declare that they have no known competing financial interests or personal relationships that could have appeared to influence the work reported in this paper.

## Appendix Supplementary materials

Image, application 1
