# Supplementary material for: Lifestyle factors and DNA methylation-based aging clocks: cross-sectional and longitudinal associations in the Singapore diet and healthy aging cohort
Source: J Prev Alzheimers Dis. 2026 Feb 27;13(4):100522. doi: 10.1016/j.tjpad.2026.100522 (PMC12964021; doi:10.1016/j.tjpad.2026.100522)
Supplement: Supplementary file 1 [file mmc1.docx]

| **Independent Variable** | **PCGrimAgeDev Cohen's d [95% CI]** | **PCGrimAgeDev p value** | **DunedinPACE Cohen's d [95% CI]** | **DunedinPACE p value** |
| --- | --- | --- | --- | --- |
| Weekly feel sleepy during the day | 0.02 [-0.13, 0.17] | 0.81 | -0.01 [-0.18, 0.16] | 0.9 |
| Daily feel sleepy during the day | 0.09 [-0.11, 0.30] | 0.38 | 0.07 [-0.17, 0.30] | 0.58 |
| Weekly have a bowel movement within 10 minutes | 0.20 [-0.07, 0.47] | 0.15 | 0.00 [-0.31, 0.31] | 0.99 |
| Daily have a bowel movement within 10 minutes | 0.15 [-0.08, 0.38] | 0.2 | -0.03 [-0.29, 0.23] | 0.81 |
| Ever work more than 9 hours/day | 0.01 [-0.31, 0.33] | 0.93 | -0.14 [-0.50, 0.22] | 0.45 |
| Ever smoke cigarette | 1.27 [0.91, 1.62] | <0.0001 | 0.58 [0.16, 0.99] | 0.01 |
| Ever drink alcohol | 0.01 [-0.22, 0.24] | 0.93 | 0.03 [-0.24, 0.29] | 0.83 |
| Weekly feel stressed | 0.29 [0.07, 0.51] | 0.01 | 0.03 [-0.22, 0.28] | 0.8 |
| Daily feel stressed | -0.31 [-0.78, 0.16] | 0.19 | -0.37 [-0.90, 0.16] | 0.17 |
| Weekly participate in physical activities | -0.39 [-0.60, -0.18] | <0.001 | -0.24 [-0.48, -0.01] | 0.04 |
| Daily participate in physical activities | -0.21 [-0.43, 0.00] | 0.05 | -0.18 [-0.43, 0.06] | 0.14 |
| Weekly participate in social activities | -0.15 [-0.31, 0.02] | 0.08 | 0.03 [-0.15, 0.21] | 0.76 |
| Daily participate in social activities | -0.01 [-0.24, 0.22] | 0.95 | 0.07 [-0.19, 0.34] | 0.59 |
| Weekly participate in cognitively demanding activities | -0.14 [-0.31, 0.04] | 0.13 | -0.07 [-0.27, 0.13] | 0.51 |
| Daily participate in cognitively demanding activities | -0.19 [-0.36, -0.02] | 0.03 | -0.17 [-0.36, 0.03] | 0.09 |
| Weekly participate in religious/spiritual activities | 0.05 [-0.11, 0.20] | 0.56 | 0.13 [-0.05, 0.31] | 0.14 |
| Daily participate in religious/spiritual activities | 0.14 [-0.16, 0.44] | 0.37 | -0.07 [-0.41, 0.28] | 0.7 |
| Weekly expose yourself to sunlight | -0.02 [-0.33, 0.29] | 0.9 | -0.11 [-0.46, 0.24] | 0.53 |
| Daily expose yourself to sunlight | 0.05 [-0.24, 0.34] | 0.74 | -0.08 [-0.41, 0.26] | 0.65 |
| Daily brush the teeth | 0.12 [-0.66, 0.89] | 0.77 | -0.70 [-1.58, 0.18] | 0.12 |
| Weekly stop eating before you feel full | 0.03 [-0.18, 0.24] | 0.79 | 0.00 [-0.24, 0.24] | 0.99 |
| Daily stop eating before you feel full | 0.06 [-0.12, 0.23] | 0.52 | 0.05 [-0.15, 0.25] | 0.61 |
| Weekly sleep < 6 hours/day | 0.03 [-0.14, 0.21] | 0.7 | -0.04 [-0.24, 0.16] | 0.69 |
| Daily sleep < 6 hours/day | 0.02 [-0.20, 0.24] | 0.85 | 0.06 [-0.19, 0.31] | 0.65 |
| Weekly sleep > 9 hours/day | 0.23 [-0.13, 0.59] | 0.22 | 0.29 [-0.12, 0.70] | 0.16 |
| Daily sleep > 9 hours/day | 0.31 [-0.04, 0.65] | 0.08 | 0.29 [-0.10, 0.69] | 0.14 |

Supplementary Table 1. Cross-sectional associations of 15 lifestyles and GrimAge AgeDev and pace of ageing adjusted for age and sex

| **Independent Variable** | **PCGrimAgeDev Cohen's d [95% CI]** | **PCGrimAgeDev p value** | **DunedinPACE Cohen's d [95% CI]** | **DunedinPACE p value** |
| --- | --- | --- | --- | --- |
| Weekly feel sleepy during the day | 0.01 [-0.12, 0.14] | 0.9 | -0.03 [-0.19, 0.14] | 0.77 |
| Daily feel sleepy during the day | 0.00 [-0.17, 0.18] | 0.96 | 0.05 [-0.18, 0.28] | 0.66 |
| Weekly have a bowel movement within 10 minutes | 0.00 [-0.24, 0.23] | 0.98 | -0.01 [-0.32, 0.30] | 0.95 |
| Daily have a bowel movement within 10 minutes | -0.01 [-0.21, 0.19] | 0.93 | -0.05 [-0.31, 0.21] | 0.71 |
| Ever work more than 9 hours/day | -0.05 [-0.32, 0.23] | 0.74 | -0.07 [-0.43, 0.28] | 0.7 |
| Ever smoke cigarette | 1.45 [1.13, 1.77] | <0.0001 | 0.63 [0.22, 1.05] | 0.003 |
| Ever drink alcohol | -0.09 [-0.30, 0.11] | 0.36 | -0.02 [-0.28, 0.24] | 0.88 |
| Weekly feel stressed | 0.17 [-0.02, 0.36] | 0.07 | 0.02 [-0.22, 0.27] | 0.85 |
| Daily feel stressed | -0.22 [-0.62, 0.19] | 0.29 | -0.29 [-0.82, 0.23] | 0.27 |
| Weekly participate in physical activities | -0.22 [-0.40, -0.04] | 0.02 | -0.15 [-0.38, 0.08] | 0.21 |
| Daily participate in physical activities | -0.06 [-0.25, 0.12] | 0.52 | -0.09 [-0.33, 0.15] | 0.46 |
| Weekly participate in social activities | -0.06 [-0.19, 0.08] | 0.43 | 0.06 [-0.12, 0.24] | 0.51 |
| Daily participate in social activities | -0.02 [-0.22, 0.18] | 0.85 | 0.03 [-0.22, 0.29] | 0.79 |
| Weekly participate in cognitively demanding activities | -0.11 [-0.26, 0.05] | 0.18 | 0.01 [-0.19, 0.20] | 0.94 |
| Daily participate in cognitively demanding activities | -0.16 [-0.31, -0.01] | 0.04 | -0.12 [-0.31, 0.07] | 0.21 |
| Weekly participate in religious/spiritual activities | 0.03 [-0.11, 0.16] | 0.68 | 0.13 [-0.04, 0.31] | 0.14 |
| Daily participate in religious/spiritual activities | -0.02 [-0.28, 0.24] | 0.86 | -0.18 [-0.52, 0.16] | 0.29 |
| Weekly expose yourself to sunlight | 0.05 [-0.22, 0.31] | 0.72 | -0.03 [-0.37, 0.32] | 0.87 |
| Daily expose yourself to sunlight | 0.15 [-0.11, 0.40] | 0.26 | 0.06 [-0.27, 0.39] | 0.74 |
| Daily brush the teeth | 0.20 [-0.46, 0.86] | 0.55 | -0.57 [-1.42, 0.29] | 0.19 |
| Weekly stop eating before you feel full | 0.08 [-0.10, 0.26] | 0.37 | 0.03 [-0.20, 0.26] | 0.78 |
| Daily stop eating before you feel full | 0.13 [-0.01, 0.28] | 0.07 | 0.07 [-0.12, 0.27] | 0.45 |
| Weekly sleep < 6 hours/day | -0.01 [-0.16, 0.14] | 0.89 | -0.05 [-0.25, 0.15] | 0.63 |
| Daily sleep < 6 hours/day | 0.04 [-0.15, 0.23] | 0.69 | 0.08 [-0.17, 0.32] | 0.55 |
| Weekly sleep > 9 hours/day | 0.26 [-0.05, 0.57] | 0.1 | 0.25 [-0.15, 0.65] | 0.23 |
| Daily sleep > 9 hours/day | 0.16 [-0.14, 0.46] | 0.3 | 0.16 [-0.23, 0.55] | 0.42 |

Supplementary Table 2. Cross-sectional associations of 15 lifestyles and GrimAge AgeDev and pace of ageing adjusted for age, sex, smoking status, BMI, education and PCA of 7 cell compositions.

| **Independent Variable** | **PCGrimAgeDev Cohen's d [95% CI]** | **PCGrimAgeDev p value** | **DunedinPACE Cohen's d [95% CI]** | **DunedinPACE p value** |
| --- | --- | --- | --- | --- |
| Weekly participate in physical activities | 0.32 [-0.34, 0.98] | 0.34 | 0.10 [-0.54, 0.75] | 0.76 |
| Daily participate in physical activities | 0.25 [-0.40, 0.89] | 0.46 | 0.14 [-0.51, 0.78] | 0.68 |
| Weekly participate in social activities | -0.19 [-0.60, 0.23] | 0.38 | -0.14 [-0.55, 0.27] | 0.5 |
| Daily participate in social activities | -0.46 [-1.25, 0.33] | 0.25 | -0.10 [-0.88, 0.69] | 0.81 |
| Weekly participate in cognitively demanding activities | 0.14 [-0.33, 0.61] | 0.55 | -0.18 [-0.64, 0.28] | 0.45 |
| Daily participate in cognitively demanding activities | 0.19 [-0.26, 0.65] | 0.41 | -0.18 [-0.63, 0.27] | 0.43 |
| Weekly participate in religious/spiritual activities | 0.31 [-0.10, 0.73] | 0.14 | 0.12 [-0.30, 0.53] | 0.58 |
| Daily participate in religious/spiritual activities | 0.38 [-0.34, 1.10] | 0.3 | 0.04 [-0.68, 0.75] | 0.92 |
| Weekly expose yourself to sunlight | -0.29 [-1.13, 0.55] | 0.5 | -0.06 [-0.90, 0.78] | 0.89 |
| Daily expose yourself to sunlight | -0.65 [-1.40, 0.10] | 0.09 | 0.27 [-0.49, 1.02] | 0.49 |
| Daily brush the teeth | -0.57 [-2.52, 1.37] | 0.56 | 0.00 [-1.98, 1.99] | 1 |
| Weekly stop eating before you feel full | 0.53 [-0.28, 1.34] | 0.2 | 0.11 [-0.70, 0.91] | 0.79 |
| Daily stop eating before you feel full | 0.42 [0.01, 0.83] | 0.049 | 0.26 [-0.15, 0.67] | 0.22 |
| Weekly sleep < 6 hours/day | 0.08 [-0.43, 0.60] | 0.76 | 0.17 [-0.34, 0.68] | 0.51 |
| Daily sleep < 6 hours/day | 0.05 [-0.49, 0.59] | 0.85 | -0.06 [-0.60, 0.47] | 0.82 |
| Weekly sleep > 9 hours/day | 0.33 [-0.51, 1.16] | 0.44 | -0.53 [-1.36, 0.29] | 0.21 |
| Daily sleep > 9 hours/day | 0.50 [-0.21, 1.22] | 0.17 | 0.10 [-0.61, 0.80] | 0.79 |
| Weekly feel sleepy during the day | -0.04 [-0.45, 0.37] | 0.84 | -0.21 [-0.61, 0.19] | 0.31 |
| Daily feel sleepy during the day | 0.19 [-0.45, 0.83] | 0.55 | -0.08 [-0.70, 0.55] | 0.81 |
| Weekly have a bowel movement within 10 minutes | -0.71 [-1.75, 0.34] | 0.19 | -0.56 [-1.59, 0.47] | 0.29 |
| Daily have a bowel movement within 10 minutes | -0.28 [-1.18, 0.61] | 0.54 | -0.08 [-0.96, 0.80] | 0.86 |
| Ever work more than 9 hours/day | 0.22 [-0.68, 1.12] | 0.64 | -0.07 [-0.96, 0.82] | 0.88 |
| Ever smoke cigarette | 1.08 [-0.13, 2.29] | 0.08 | -0.41 [-1.59, 0.76] | 0.49 |
| Ever drink alcohol | -0.34 [-1.11, 0.42] | 0.38 | 0.22 [-0.54, 0.98] | 0.57 |
| Weekly feel stressed | -0.24 [-0.84, 0.36] | 0.43 | -0.33 [-0.93, 0.26] | 0.27 |
| Daily feel stressed | 0.08 [-1.33, 1.48] | 0.92 | -0.17 [-1.55, 1.21] | 0.81 |

Supplementary Table 3. Longitudinal associations of 15 lifestyles and GrimAge AgeDev and pace of ageing adjusted for age and sex

| **Independent Variable** | **PCGrimAgeDev Cohen's d [95% CI]** | **PCGrimAgeDev p value** | **DunedinPACE Cohen's d [95% CI]** | **DunedinPACE p value** |
| --- | --- | --- | --- | --- |
| Weekly participate in physical activities | 0.59 [-0.06, 1.24] | 0.08 | 0.12 [-0.60, 0.84] | 0.75 |
| Daily participate in physical activities | 0.55 [-0.10, 1.19] | 0.1 | 0.15 [-0.56, 0.86] | 0.68 |
| Weekly participate in social activities | -0.08 [-0.48, 0.32] | 0.7 | -0.15 [-0.58, 0.29] | 0.51 |
| Daily participate in social activities | -0.20 [-1.01, 0.61] | 0.63 | -0.09 [-0.97, 0.79] | 0.83 |
| Weekly participate in cognitively demanding activities | 0.14 [-0.31, 0.59] | 0.55 | -0.10 [-0.59, 0.39] | 0.69 |
| Daily participate in cognitively demanding activities | 0.19 [-0.26, 0.65] | 0.41 | -0.07 [-0.56, 0.43] | 0.79 |
| Weekly participate in religious/spiritual activities | 0.33 [-0.10, 0.76] | 0.14 | 0.27 [-0.19, 0.74] | 0.25 |
| Daily participate in religious/spiritual activities | 0.17 [-0.57, 0.91] | 0.66 | 0.23 [-0.58, 1.04] | 0.58 |
| Weekly expose yourself to sunlight | -0.08 [-0.95, 0.79] | 0.85 | -0.03 [-0.97, 0.90] | 0.94 |
| Daily expose yourself to sunlight | -0.29 [-1.07, 0.49] | 0.47 | 0.31 [-0.54, 1.16] | 0.48 |
| Daily brush the teeth | -0.35 [-2.23, 1.53] | 0.72 | 0.24 [-1.86, 2.34] | 0.82 |
| Weekly stop eating before you feel full | 0.53 [-0.28, 1.33] | 0.2 | 0.01 [-0.86, 0.88] | 0.98 |
| Daily stop eating before you feel full | 0.26 [-0.17, 0.68] | 0.25 | 0.32 [-0.14, 0.79] | 0.18 |
| Weekly sleep < 6 hours/day | 0.27 [-0.23, 0.77] | 0.29 | 0.18 [-0.36, 0.72] | 0.52 |
| Daily sleep < 6 hours/day | 0.00 [-0.55, 0.54] | 0.99 | -0.02 [-0.61, 0.58] | 0.95 |
| Weekly sleep > 9 hours/day | -0.15 [-1.01, 0.70] | 0.72 | -0.48 [-1.39, 0.43] | 0.3 |
| Daily sleep > 9 hours/day | 0.56 [-0.13, 1.26] | 0.12 | 0.14 [-0.62, 0.91] | 0.72 |
| Weekly feel sleepy during the day | -0.06 [-0.45, 0.33] | 0.76 | -0.22 [-0.64, 0.20] | 0.3 |
| Daily feel sleepy during the day | 0.13 [-0.49, 0.76] | 0.67 | 0.03 [-0.65, 0.70] | 0.94 |
| Weekly have a bowel movement within 10 minutes | -0.67 [-1.71, 0.36] | 0.2 | -0.71 [-1.83, 0.42] | 0.22 |
| Daily have a bowel movement within 10 minutes | -0.19 [-1.09, 0.71] | 0.68 | -0.23 [-1.21, 0.75] | 0.64 |
| Ever work more than 9 hours/day | 0.31 [-0.56, 1.19] | 0.48 | -0.21 [-1.16, 0.75] | 0.67 |
| Ever smoke cigarette | 0.41 [-0.86, 1.69] | 0.53 | -0.18 [-1.48, 1.12] | 0.78 |
| Ever drink alcohol | -0.24 [-0.97, 0.50] | 0.53 | 0.41 [-0.40, 1.21] | 0.32 |
| Weekly feel stressed | -0.23 [-0.81, 0.35] | 0.44 | -0.33 [-0.95, 0.30] | 0.31 |
| Daily feel stressed | -0.24 [-1.65, 1.18] | 0.74 | 0.10 [-1.43, 1.63] | 0.9 |

Supplementary Table 4. Longitudinal associations of 15 lifestyles and GrimAge AgeDev and pace of ageing adjusted for age, sex, smoking status, BMI, education and PCA of 7 cell compositions.
